# Coupled enhancer and coding sequence evolution of a homeobox gene shaped leaf diversity

**DOI:** 10.1101/gad.290684.116

**Published:** 2016-11-01

**Authors:** Francesco Vuolo, Remco A. Mentink, Mohsen Hajheidari, C. Donovan Bailey, Dmitry A. Filatov, Miltos Tsiantis

**Affiliations:** 1 Department of Comparative Development and Genetics, Max Planck Institute for Plant Breeding Research, 50829 Cologne, Germany;; 2 Department of Biology, New Mexico State University, Las Cruces, New Mexico 88003, USA;; 3 Department of Plant Sciences, University of Oxford, Oxford OX1 3RB, United Kingdom

**Keywords:** compound leaf, leaf development, plant homeobox gene, regulatory evolution

## Abstract

Here we investigate mechanisms underlying the diversification of biological forms using crucifer leaf shape as an example. We show that evolution of an enhancer element in the homeobox gene *REDUCED COMPLEXITY* (*RCO*) altered leaf shape by changing gene expression from the distal leaf blade to its base. A single amino acid substitution evolved together with this regulatory change, which reduced RCO protein stability, preventing pleiotropic effects caused by its altered gene expression. We detected hallmarks of positive selection in these evolved regulatory and coding sequence variants and showed that modulating RCO activity can improve plant physiological performance. Therefore, interplay between enhancer and coding sequence evolution created a potentially adaptive path for morphological evolution.

## Abstract (precis)

In this study, Vuolo et al. investigate the mechanisms underlying the genetic basis for morphological diversity in leaf shape. They show that evolution of an enhancer element in the homeobox gene *REDUCED COMPLEXITY* (*RCO*) altered leaf shape by changing gene expression from the distal leaf blade to its base.

Understanding the genetic basis for evolutionary change is a fundamental problem in biology. Morphological diversity is often underpinned by *cis*-regulatory divergence of developmental genes and consequent spatiotemporal modification of their expression (Gompel et al. 2005; Hay and Tsiantis 2006; Prud'homme et al. 2006; Carroll 2008; Chan et al. 2010; Frankel et al. 2011; Studer et al. 2011; Arnoult et al. 2013; Rast-Somssich et al. 2015; Indjeian et al. 2016). However, the origin of specific *cis*-regulatory elements underlying morphological diversity is still poorly understood (Rebeiz et al. 2015). For example, it is unclear whether such *cis* elements tend to arise de novo from rapidly evolving sequences or through the co-option of existing conserved regulatory sequences (Rebeiz et al. 2011; Boyd et al. 2015; Villar et al. 2015). Furthermore, it has not been investigated whether and how coding sequences evolve in concert with regulatory changes to optimize gene function in a new expression domain. Finally, links between regulatory changes underlying morphological change and organismal physiology and fitness remain scarce.

Plant leaves present a useful genetic model to tackle these questions because they show substantial morphological variation (Shleizer-Burko et al. 2011; Bar and Ori 2014) and have considerable eco–physiological importance as the major site of photosynthetic carbon fixation in terrestrial ecosystems (Givnish 1978). The *REDUCED COMPLEXITY* (*RCO*) gene played a key role in leaf shape diversification in the crucifer family (Sicard et al. 2014; Vlad et al. 2014), to which the reference plant *Arabidopsis thaliana* belongs. *RCO* arose through gene duplication and encodes a class I homeobox leucine zipper protein. Its function was discovered in *Cardamine hirsuta*, where it acts to divide the leaf into distinct leaflets by locally repressing growth at the leaf margin, creating a complex shape. This species-specific activity of *RCO* arose by neofunctionalization following gene duplication of its ancestral paralog, *LMI1*, which is conserved in seed plants. Specifically, *RCO* acquired a novel expression domain within the growth zone at the base of the leaf, where growth repression—a conserved function of the RCO/LMI protein—exerts a greater effect on leaf shape (Fig. 1A). *RCO* was secondarily lost in *A. thaliana*, leading to leaf simplification, and its reintroduction in the *A. thaliana* genome was sufficient to increase leaf complexity (Vlad et al. 2014). Thus, *RCO* is a large effect gene underlying morphological diversity and offers an excellent system to explore the causes and consequences of morphological evolution. Here, we identify the specific molecular events underpinning the evolution of *RCO* function and provide evidence that modulating RCO activity can improve plant physiological performance.

**Figure 1.**
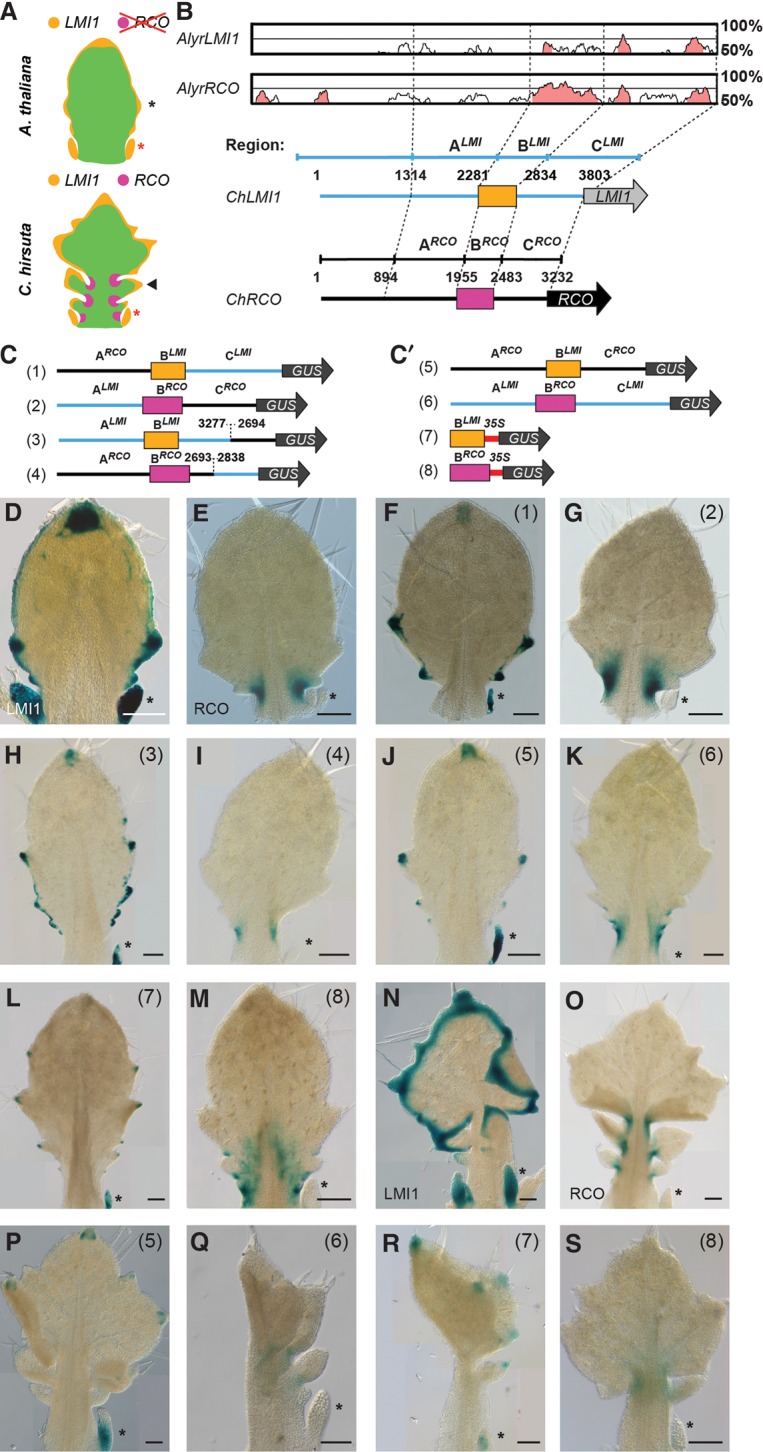
*ChRCOenh*^*500*^ is sufficient to drive proximal expression in the leaf lamina and evolved via modification of an enhancer driving distal expression in the leaf lamina. (*A*) Cartoon depicting leaf primordia of *A. thaliana* (*top*; black star indicates serration) and *C. hirsuta* (*bottom*; black arrowhead indicates leaflet). Red stars indicate stipules located at the leaf base. *LMI1* (orange) and *RCO* (magenta) expression is shown. (*B*, *top*) mVISTA plot of *Arabidopsis lyrata LMI1* and *RCO* upstream sequence aligned to *C. hirsuta RCO*, indicating sequence conservation (50%–100%) (mVISTA alignments with additional species are shown in Supplemental Fig. 1). Pink indicates conserved nucleotide sequences (CNSs; identity >70% within 100 base pairs [bp]). (*Bottom*) Subdivision of *LMI1* and *RCO* upstream sequences into regions A, B, and C based on CNSs. *ChLMI1* and *ChRCO* gene models (blue and black lines, respectively) with the CNS enhancer element of ∼500 bp indicated. (Orange) B^*LM1*^; (magenta) B^*RCO*^. (*C*,*C*′) Chimeric GUS reporter constructs (1–8) used to identify critical *cis*-regulatory elements in *ChLMI1* and *ChRCO* upstream regions. (*D*–*S*) Representative *A. thaliana* (*D*–*M*) and *C. hirsuta* (*N*–*S*) GUS-stained leaves carrying reporter constructs depicted in *C* and *C*′ or full-length 3.8-kb *ChLMI1* (*D*,*N*) and 3.2-kb *ChRCO* (*E*,*O*) upstream regions (see also Vlad et al. 2014). In each image, the construct used is indicated (1–8). The *A. thaliana* leaf depicted in *L* is more mature, hence the higher number of serrations. Each image contains at least one stipule (black star) to visualize the presence (*LMI1*) or absence (*RCO*) of expression. In each case, at least two independent T2 lines were analyzed with *n* > 5. Bar, 100 μm.

## Results and Discussion

To understand whether discrete enhancer sequences explain the difference in expression between *RCO* and its ancestral paralog, *LMI1*, we analyzed the upstream sequences of *LMI1* and *RCO* using transgenic assays. We first investigated whether discrete enhancer sequences are sufficient to explain the evolutionary shift in *RCO* expression with respect to its paralog, *LMI1*, and what their origin might be. We reasoned that if such enhancer elements exist in *RCO*, their introduction in *LMI1* via chimeric constructs should recapitulate evolution and convert the ancestral distal expression pattern into the proximal one of *RCO*. We first defined upstream noncoding DNA fragments of *RCO* and *LMI1* that were sufficient to drive reporter gene expression in the proximal and distal domains of the leaf lamina that characterize each gene (Supplemental Fig. 2A–E; Supplemental Table 3). Subsequently, we assayed the expression pattern of chimeric reporter genes where three individual segments (regions A, B, and C) of *RCO* and *LMI1* upstream sequences were swapped between the two genes. (Fig. 1B–C′; Supplemental Table 3). We conducted these reporter gene assays in *A. thaliana*, as the upstream regulatory regions of *C. hirsuta LMI1* and *RCO* recapitulate their respective distal and proximal expression patterns in the leaf lamina of *A. thaliana* (Fig. 1D,E; Supplemental Fig. 2B,D; Vlad et al. 2014).

These chimeric reporters had a binary readout: Each reporter yielded either the *LMI1-*type or the *RCO*-type expression pattern (Fig. 1F–K). The *LMI1-*type pattern was defined by expression in stipules and hydathodes, with weaker expression in the leaf margin. In comparison with this, the *RCO*-type pattern was expressed only at the base of the leaf blade. These observations indicated that specific sequences contributing to *LMI1* expression might have been modified through evolution to produce the *RCO* expression pattern. In support of this idea, exchanging region B^*RCO*^ for the corresponding *LMI1* sequence converted the *LMI1* expression pattern into the *RCO* pattern in both *A. thaliana* and the endogenous *C. hirsuta* context (Fig. 1K,Q). Conversely, introducing region B^*LMI*^ into the *RCO* sequence resulted in an *LMI1* expression pattern in both the *A. thaliana* and *C. hirsuta* contexts (Fig. 1J,P). Moreover, a reporter containing only region B^*LMI*^ or B^*RCO*^ coupled to a 50-base-pair (bp) CaMV 35S minimal promoter was sufficient to drive specific *LMI1-*type or *RCO-*type expression in *A. thaliana* (Fig. 1C′,L,M) and, in the case of B^*RCO*^, also in *C. hirsuta* (Fig. 1R,S). Notably, the corresponding region B^*LMI*^ from *Aethionema arabicum*, an early divergent crucifer, is necessary for correct *LMI1* gene expression (Supplemental Fig. 2G–H), also indicating that it was already functional before the divergence of *Aethionema* from other crucifers (Vlad et al. 2014). Thus, the 500-bp region B^*LMI*^ or B^*RCO*^ has a key function in determining the expression pattern of its respective downstream gene. Consequently, we call this region the *RCO* or *LMI1* 500-bp enhancer (*ChRCOenh*^*500*^ or *ChLMI1enh*^*500*^).

**Figure 2.**
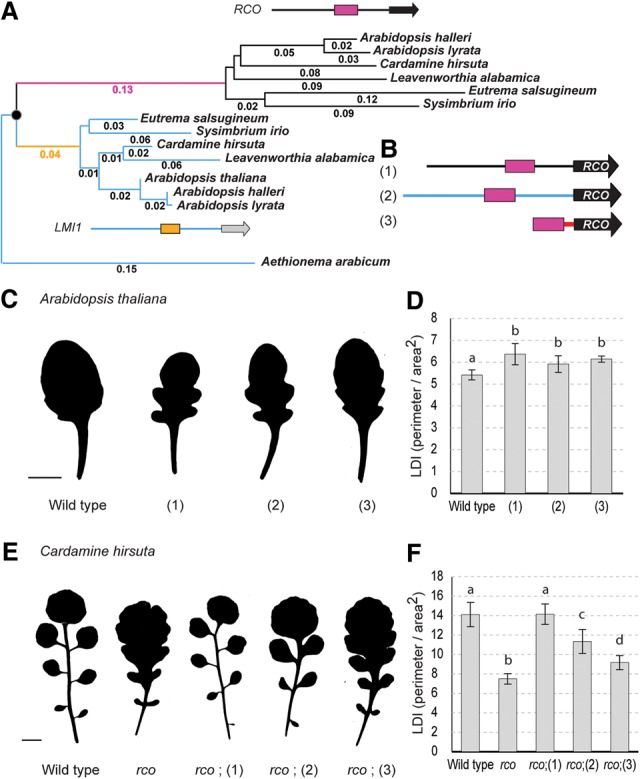
*ChRCOenh*^*500*^ evolved nonneutrally and is sufficient to increase leaf complexity when driving *RCO* in *A. thaliana* and *C. hirsuta*. (*A*) The *RCO* enhancer (magenta; depicted on a black gene model) experienced an increased base substitution rate (likelihood ratio test: ΔlnL = 9.64; *P* < 0.005) compared with the *LMI1* enhancer (yellow; depicted on a blue gene model), consistent with positive selection (see the Supplemental Material for detailed analysis). Base substitution rates are indicated next to individual branches. (*B*) Constructs (1–3) used to express RCO in the *RCO* domain contain the upstream sequence of *ChLMI1* (blue line), *ChRCO* (black line), or the *CaMV 35S* minimal promoter (red line) with enhancer B^*RCO*^ (magenta). (*C*,*E*) Representative leaf 8 silhouettes from *A. thaliana* (*C*) and *C. hirsuta* (*E*) wild-type and transgenic plants. Bar, 1 cm. (*D*,*F*) Leaf dissection index ([LDI] perimeter/area^2^) calculated from *A. thaliana* (*D*) and *C. hirsuta* (*F*) leaf 8 silhouettes. Graphs indicate average LDI and standard deviation. Letters indicate significant differences between groups as indicated by ANOVA and post-hoc Tukey's test. *P* < 0.01. For constructs 2 and 3, at least three independent T2 lines were analyzed with *n* > 12.

To test to what degree gene expression conferred by *ChRCOenh*^*500*^ is phenotypically relevant, we used it to express the *RCO*-coding sequence both in the *LMI1* regulatory sequence context and using a 35S minimal promoter (Supplemental Table 3). Strikingly, both constructs increased leaf complexity in *A. thaliana* and rescued the *rco* mutant leaf phenotype in *C. hirsuta* (Fig. 2B–F). These findings demonstrate that *ChRCOenh*^*500*^ is necessary and sufficient to drive RCO function and that this sequence imparts morphologically relevant transcriptional information even in the context of a heterologous promoter. *ChRCOenh*^*500*^ must interact with additional sequences to ensure the correct level of *RCO* transcription, as the 2.3-kb fragment drives higher expression (Figs. 1M, 2C–F; Supplemental Fig. 2E). In summary, *ChRCOenh*^*500*^ recapitulates the *RCO* expression pattern, and its activity is sufficient to increase leaf complexity when transferred between two reproductively isolated species that diverged ∼30 million years ago (Vlad et al. 2014). Our findings demonstrate that a specific enhancer element in *LMI1*, which directs distal gene expression, neofunctionalized in the *RCO* duplicate gene to yield a novel expression pattern at the leaf base, resulting in a novel leaf form.

Next, we investigated the evolutionary forces that led to the diversification of *RCOenh*^*500*^ from its *LMI1* counterpart by comparing their sequence divergence patterns in a phylogenetic framework. We observed a significantly higher base substitution rate for this enhancer within the *RCO* clade than within the *LMI1* clade (Fig. 2A). Using a modified branch site likelihood model adapted for noncoding regions (Wong and Nielsen 2004), we demonstrated that this accelerated evolution of *RCOenh*^*500*^ likely reflects the action of positive selection. These analyses, coupled with our functional data (Fig. 1B–S), are consistent with the idea that positive selection helped shape the *RCO* expression domain via acting on *ChRCOenh*^*500*^.

**Figure 3.**
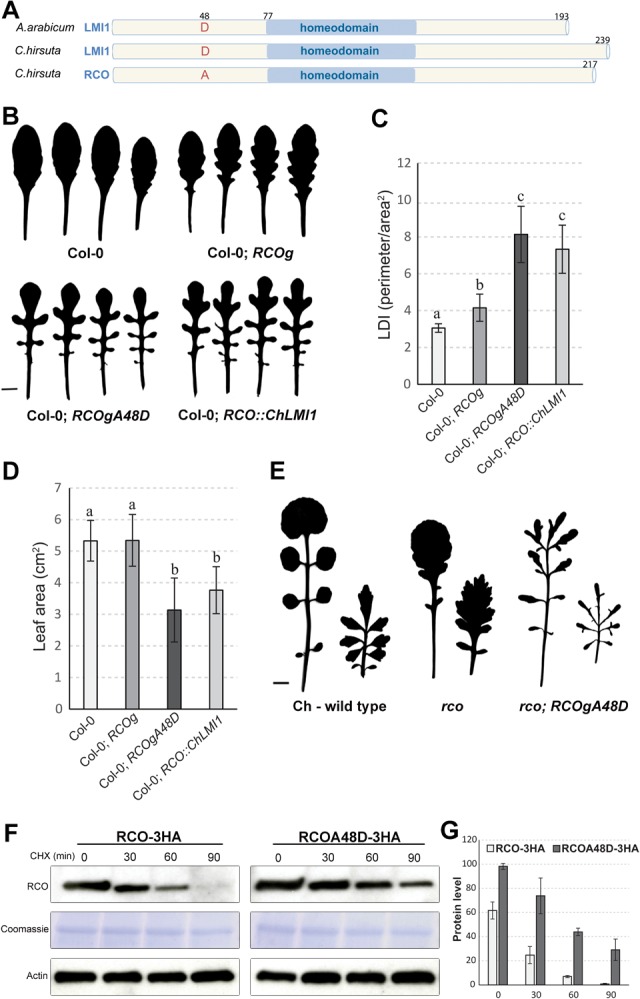
Positive selection dampened RCO protein function via a D > A transition that decreased protein stability. (*A*) Structure of LMI1/RCO protein in *A. arabicum* and *C. hirsuta*. (*B*–*D*) Silhouettes (*B*), leaf dissection index (*C*), and leaf area (*D*) of leaves 7 to 10 of *A. thaliana* wild-type, *RCOg*, *RCOgA48D*, and *RCO::ChLMI1* plants. Error bars represent standard deviation based on at least 15 independent T1 lines. Letters indicate significant differences between groups as indicated by ANOVA and post-hoc Tukey's test. *P* < 0.01. (*E*) Silhouettes of rosette and cauline leaves of *C. hirsuta* wild type, *rco*, and *rco; RCOgA48D*. (*F*,*G*) Quantitative analysis of RCO-3HA and RCOA48D-3HA protein levels after cyclohexamide (CHX) treatment at the indicated time points. (*F*) Coomassie blue staining (SDS-PAGE slice containing a 57-kDa band) and anti-Actin immunoblotting indicate equal loading. (*G*) Protein level quantification of samples shown in *F*. Error bars represent standard deviation of the mean protein level using three biological replicates.

RCO/LMI1 proteins are potent growth repressors, and their broad expression results in miniature plants (Vlad et al. 2014). This raises the question of whether *RCO* enhancer evolution involved concomitant coding sequence diversification to alleviate potentially pleiotropic effects resulting from altered *RCO* expression. To address this question, we analyzed *RCO*-coding sequence diversification patterns from seven species. A phylogeny-based maximum likelihood ratio test (Yang 2007) identified signals of positive selection centered on the alanine and tyrosine residues at positions 48 (A48) and 56 (Y56), N-terminal to the homeodomain (Fig. 3A; Supplemental Table 1). To test the functional importance of these D48A and S56Y changes in the RCO protein, we generated *A. thaliana* plants expressing modified *RCO* genes (*RCOgA48D/RCOgY56S/RCOgA48D-Y56S*) where the native promoter drives RCO with the A48D and Y56S mutations individually or in combination. The leaf phenotype of plants expressing *RCOY56S* was indistinguishable from plants expressing *RCO* (Supplemental Fig. 3). However, plants expressing *RCOA48D* or *RCOA48D-Y56S* had more dissected leaves (Supplemental Fig. 3) and resembled plants expressing *LMI1* in the *RCO* domain (Fig. 3B,C), indicating that the A48D mutation has a major effect on leaf form. The stronger effect of the A48D versus the Y56S change in RCO is consistent with a greater contrast between the properties of the derived and ancestral amino acids: Alanine (A) is nonpolar, neutral, and hydrophobic, and aspartic acid (D) is polar, acidic, and hydrophilic, whereas tyrosine (Y) and serine (S) are very similar. However, the possibility that the Y56S mutation might cause phenotypic effects under different growth conditions cannot be excluded.

The increased leaf complexity in transgenic *RCOgA48D* and *RCO::ChLMI1* plants was accompanied by a significant decrease in leaf area (Fig. 3B–D) compared with *RCOg* plants. Thus, the RCOA48D and LMI1 proteins are more potent than RCO, resulting in not only altered leaf shape but also compromised organ growth when expressed in the *RCO* domain. In contrast, the native RCO protein changes *A. thaliana* leaf shape without incurring an organ growth penalty (Fig. 3B–D). RCOA48D showed consistently higher potency than RCO in the endogenous *C. hirsuta* context: It rescued the *rco* mutant phenotype more effectively, increased wild-type leaf complexity, and reduced leaf size (Fig. 3E; Supplemental Fig. 4A–D). Reduction in cell size contributes to the reduced leaf surface of *RCOgA48D* (Supplemental Fig. 5), indicating that RCO/LMI1 proteins may repress growth at the whole-organ level by repressing cell growth. Taken together, these observations indicate that diversification of gene expression after duplication of the ancestral *LMI1* gene entailed a risk of pleiotropic effects, detrimental to growth. We propose that these effects were counteracted by the D48A amino acid change, which dampened RCO protein potency. Two lines of evidence indicate that this dampening involved reduced protein stability. First, HA-tagged RCOA48D (RCOA48D-3HA) accumulated to higher levels in transgenic plants than HA-tagged RCO (RCO-3HA) (Supplemental Fig. 6A–C). Second, the degradation rate of RCO-3HA is higher than RCOA48D-3HA following de novo protein synthesis inhibition by cycloheximide (Fig. 3F,G). These findings highlight the importance of coordinated coding and regulatory sequence evolution for morphological variation. They also indicate that coupling protein and *cis*-regulatory evolution (Prud'homme et al. 2006; Stern and Orgogozo 2008; Chan et al. 2010; Frankel et al. 2011; Indjeian et al. 2016) can effectively minimize the pleiotropic effects of mutations in developmental genes. Notably, regulatory sequence variation in humans may minimize the detrimental effects of deleterious coding sequence mutations in highly expressed haplotypes (Lappalainen et al. 2011). Thus, coevolution of enhancers with their cognate coding sequences may be of broad significance across complex eukaryotes and at different evolutionary scales.

**Figure 4.**
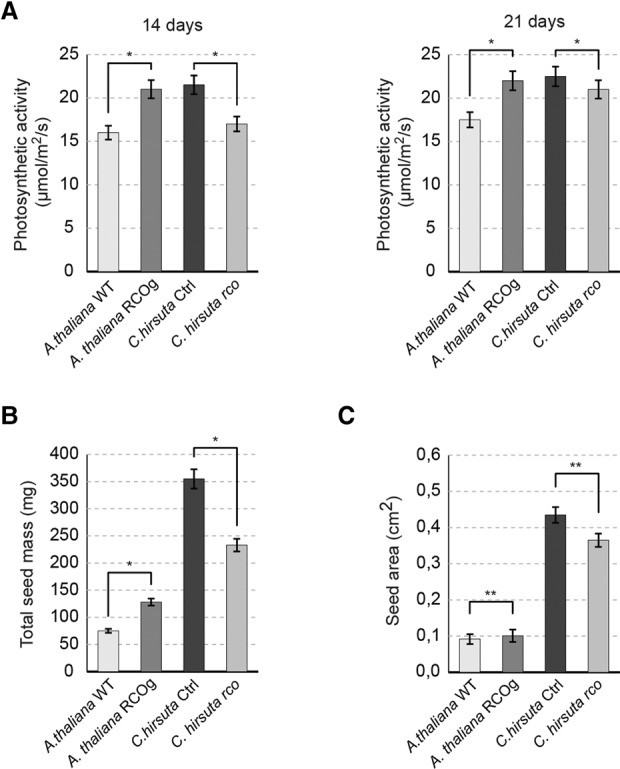
Alterations in *RCO* activity influence plant physiological performance. (*A*–*C*) Level of photosynthesis (CO_2_ absorbed per second, normalized to rosette area) after 14 and 21 d of growth (*A*), total seed mass per plant (*B*), and seed area (*C*) for *A. thaliana* wild type and *RCOg* and *C. hirsuta* control (Ctrl) and *rco*. Five replicates were measured per genotype at each time point to analyze photosynthetic activity. Total seed mass was estimated from five plants per genotype. The seed area was obtained from 50 seeds per genotype derived from five plants. Error bars represent standard deviation. A *t*-test was used to calculate significance. (N.S.) Not significant; (*) *P* < 0.05; (**) *P* < 0.01; (***) *P* < 0.001.

The hallmarks of positive selection in *RCO* indicate that it may have evolved adaptively. To investigate this hypothesis, we tested whether changes in *RCO* activity affected plant physiological performance. *rco* mutants showed reduced CO_2_ fixation (Fig. 4A), and introducing *RCO* into *A. thaliana* (*RCOg* genotype) was sufficient to increase CO_2_ fixation by 20%–25% (Fig. 4A). Furthermore, *RCO* positively influenced seed yield in both *C. hirsuta* and *A. thaliana* (Fig. 4B,C). *RCO* has a restricted expression pattern during plant development (Vlad et al. 2014) and is not expressed in the nutritive endosperm tissue of the seed but influences its size (Supplemental Fig. 7A–I). Therefore, the stimulatory effects of *RCO* on photosynthesis may ultimately influence resource allocation to seeds. Taken together, these findings strengthen the hypothesis that *RCO* evolved adaptively. These findings do not imply that complex leaves are superior to simple ones, as both forms occur readily in nature. Rather, they highlight the potential for complex leaves to perform better under certain conditions that may have been relevant during the evolutionary history of the species that we studied here (Piazza et al. 2010). Complex leaves are more prevalent under lower mean annual temperatures (Royer and Wilf 2006). Therefore, leaf margin geometry may influence the interplay between temperature and photosynthesis. The effects of *RCO* and leaf complexity on photosynthesis are unlikely to involve stomatal density (Supplemental Fig. 7J) but might arise from conditional improvement in some or all of the following processes: gas exchange, due to reduced air boundary layer thickness, as in other complex leaves (Royer and Wilf 2006); light capture, owing to reduced shading by older leaves (Niklas 1988); and hydraulics, owing to vasculature properties in a complex blade (Dengler and Kang 2001). Notably, okra-leaf cotton shows increased photosynthesis and leaf complexity (Wells et al. 1986) together with altered *LMI1* expression (Chang et al. 2016). Therefore, our findings highlight the potential to improve photosynthesis via modulating RCO/LMI1 activity.

In conclusion, we show that neofunctionalization of an enhancer element coupled with targeted coding sequence diversification was instrumental in generating an altered leaf form with potential physiological benefits while at the same time minimizing pleiotropic effects. This type of trade-off—where molecular level functions are dampened to facilitate development of tissue- or organism-level traits—may be a pervasive feature of morphological evolution. For example, the activity of a key developmental enhancer in *Ciona* was found recently to be constrained by trade-offs between the specificity of gene activation and the level of transcriptional activity (Farley et al. 2015).

## Materials and methods

Plants were cultivated in growth chambers under long-day (16-h d/8-h night) or short-day (8-h d/16-h night) conditions. *A. thaliana* and *C. hirsuta* were transformed using *Agrobacterium tumefaciens* floral dip transformations as in Hay et al. (2014). Histochemical detection of β-glucoronidase activity and subsequent visualization of samples were essentially according to Bilsborough et al. (2011). Selection tests on the promoter and coding sequences were conducted using a modified likelihood ratio test and phylogenetic analysis by maximum likelihood (PAML), respectively. Protein stability was determined after treatment with cycloheximide to inhibit protein synthesis. Gas exchange assays were conducted according to the LICOR 6400 xt manufacturer's protocol. A detailed description of the Materials and Methods is in the Supplemental Material.

## Supplementary Material

Supplemental Material
