# Supplementary material for: Coupled enhancer and coding sequence evolution of a homeobox gene shaped leaf diversity
Source: Genes Dev. 2016 Nov 1;30(21):2370–5. doi: 10.1101/gad.290684.116 (PMC5131777; doi:10.1101/gad.290684.116)
Supplement: Supplemental Material [file supp_30_21_2370__index.html]

Coupled enhancer and coding sequence evolution of a homeobox gene shaped leaf diversity — Supplemental Material 

# Coupled enhancer and coding sequence evolution of a homeobox gene shaped leaf diversity

## Supplemental Material

**Files in this Data Supplement:**

- Supplemental Material.pdf
