# Supplementary material for: Coupled enhancer and coding sequence evolution of a homeobox gene shaped leaf diversity
Source: Genes Dev. 2016 Nov 1;30(21):2370–5. doi: 10.1101/gad.290684.116 (PMC5131777; doi:10.1101/gad.290684.116)
Supplement: Supplemental Material [file supp_gad.290684.116_Supplemental_Material.pdf]

Supplemental Methods

Construction of binary vectors

To understand the evolution of protein function within the Brassicaceae, the coding sequences of *ChRCO*, *ChRCOA48D, ChRCOY56S, ChRCOA48D-Y56S* and *ChLMI1*genes, which had been synthesized by Genscript (Hong Kong Ltd), were cloned downstream of the *RCO* promoter and upstream of the *RCO* terminator in the pBJ36 intermediate vector. Cassettes were transferred as *NotI* fragments into pMLBART for plant transformation. We generated chimeric constructs for *LMI* and *RCO* upstream sequences guided by VISTA analyses of a multiple alignment, on the basis of which we divided the upstream sequence in three separate blocks that were designated as A, B and C from 5' to 3'(Dubchak et al. 2000; Frazer et al. 2003). From these, Region B emerged as a plausible candidate for contributing to evolution of the *RCO* expression pattern because it contained continuous sequence blocks that are more strongly conserved in *RCO* than *LMI* sequences. We amplified and synthesized the different fragments of the promoter regions, and cloned them into an intermediary vector driving a *GUS* reporter gene or RCO coding sequence as shown in the Supplemental methods Table S4. To test for sufficiency of the 500 base pair CNS enhancer region B^{LMI1} or B^{RCO} to drive specific expression we coupled them to a 35S minimal promoter comprised by the sequence from position -46 to +8 of the 35S promoter (constructs (7) and (8) in Fig. 1, with the 35S minimal promoter indicated in red) (Benfey et al. 1990). To test for necessity we used the ancestral *LMI1* 5' upstream sequence of *Aethionema arabicum* and randomized the conserved corresponding B^{LMI} region within its native context (constructs (5) and (6) in Supplemental Fig. 2).

Agrobacterium-mediated transformation

A. thaliana and *C. hirsuta* plants (Columbia and Oxford strains respectively) were transformed by *A. tumefaciens* strain GV3101 using the floral dip method (Clough and Bent 1998; Koncz and Schell 2002). The siliques of soil-grown bolting plants were removed before transformation to reduce the number of untransformed seeds. Agrobacterium cultures grown in 500 ml YEB medium at 28°C to an OD600 of 0.8 to 1.0 were harvested by centrifugation (4°C, 10,000 g) and resuspended in infiltration medium containing 4.3 g/l MS basal salt mixture (Sigma), 1 x B5 vitamin (Sigma), 3% sucrose, 5 mM MES, 0.05μM BAP (6-benzylamino purine), and 0.005% (v/v) Silwet L77. Inflorescences of plants were submerged in the *Agrobacterium* suspension for 3-5 minutes. Subsequently, the plants were covered with plastic bags for two days adaptation. Seeds were collected in paper bags, dried and subjected to selection.

Quantitative RT-PCR

Total RNA was extracted using the RNeasy Mini kit supplied with RNase-Free DNase (Qiagen). Two micrograms of total RNA template was reverse transcribed using a transcriptor first-strand cDNA synthesis kit (SuperScript® VILO™ cDNA Synthesis Kit, Invitrogen). The reaction mixture was diluted to 100 μl, and 4 to 5 μl aliquots were used for real-time PCR assays performed with iQ Supermix (Bio-Rad) in a Bio-Rad iCycler iQ5. All quantitative RT-PCR measurements were performed with triplicates from three independent experiments. Standard curves were obtained with *UBQ10* primers (Supplemental Table 2) and used for internal sample normalization and quantification as described (User Bulletin; Applied Biosystems).

β -Glucuronidase (GUS) staining

The detection of GUS activity in reporter lines was performed as previously described (Hay and Tsiantis 2006). Briefly, plants fixated in 90% acetone were incubated overnight at 37 °C with 1 mg/ml of 5-bromo-4-chloro-3-indolyl-ß-D-glucuronic acid (X-gluc, Roth), supplemented with 2 mM ferricyanide and ferrocyanide salts. The *C. hirsuta* plants carrying *ChLMI1::GUS* full length construct were incubated at 37 °C for 2 hours, due to strong staining. Moreover, reporter constructs comprising only region B^{LMI} or B^{RCO} (constructs (7) and (8) in Fig. 1) in *C. hirsuta* were incubated with 0.1 mM of ferricyanide and ferrocyanide salts, due to weak staining. Images were obtained using a Zeiss Axio Imager.M2 light microscope and Zeiss Axiocam. For *A. thaliana*, for each independent construct at least 10 T1 individuals were observed to determine expression pattern consistency, which was also verified in T2. In *C. hirsuta*, all analyses were conducted in at least three independent T2 lines. In these experiments we did not aim to directly compare expression level of different constructs but focused on their expression pattern and effects on morphology. However, the consistently lower expression level driven by constructs harboring *ChRCOenh*^{500} and *ChLMI1enh*^{500} relative to their control constructs indicates that other elements in the *RCO* and *LMI1* upstream sequences studied here can also influence expression of these genes particularly in terms of their level.

Leaf shape analysis

All phenotypic measurements were performed using at least 15 independent T1 lines or 3 independent T2 lines grown under the same conditions. To obtain leaf silhouettes, fully developed leaves were flattened onto white paper using a clear adhesive and then digitally scanned. Leaf area and perimeter were calculated from silhouettes using ImageJ software. Leaf dissection index was then determined by using the formula [(perimeter)/(area

squared)] (McLellan and Endler 1998).

Scanning electron microscopy

Scanning electron micrographs were prepared using SUPRA 40VP (Zeiss, Heidelberg, Germany).

Analysis of nucleotide substitution rates in the *ChRCOenh*^{500} and *ChLMI1enh*^{500} regions.

To reconstruct a combined (*LMI1* + *RCO*) phylogeny for 8 species (*A. arabicum, A. halleri, A. lyrata, A. thaliana, C. hirsuta, E. salsugineum, L. alabamica* and *S. irio),* out of which *A. arabicum* and *A. thaliana* do not possess a copy of the *RCO* gene, we concatenated the alignments for all non-coding regions and 4-fold degenerate positions from the coding regions of *LMI1* and *RCO* genes. The phylogeny for the concatenated 6kb long alignment was reconstructed using maximum likelihood with Tamura-Nei substitution model (Tamura and Nei 1993), as implemented in MEGA software v6.06 (Tamura et al. 2013). Robustness of the phylogeny was tested with 1000 bootstrap replicates and topology of the bootstrap consensus tree was used for downstream analyses of substitution rates in *LMI1* and *RCO* genes. Phylogenetic trees for separate regions were reconstructed in a similar way.

Substitution rates between *LMI1* and *RCO* genes were compared using a local molecular clock model implemented in *baseml* program from the PAML package (Yang 2007). Likelihood ratio tests (LRT) were used to test the significance of difference between substitution rates in different clades. Significant difference was inferred when a model

allowing separate substitution rates in two clades fitted the data significantly better than the model forcing the clades to have the same substitution rate.

The presence of positive selection in the regulatory region was tested using a modified branch-site model (Zhang et al. 2005) adapted for testing selection in non-coding regions (Wong and Nielsen 2004). The approach is analogous to inferences of selection in coding regions based on comparisons of synonymous (Ks) and non-synonymous (Ka) substitution rates, with positive selection inferred when Ka/Ks > 1. For non-coding regions, that approach requires a comparison of substitution rates in putatively selected and neutral regions, with neutral region being either synonymous or non-coding (e.g. intronic) sites (Wong and Nielsen 2004). To test for positive selection we used likelihood ratio tests, as implemented in *HyPhy* batch file *nonCodingSelection.bf* (https://github.com/ofedrigo/TestForPositiveSelection). Positive selection at a specific ('foreground') branch of a phylogeny was inferred when a model allowing for positive selection at the foreground branch fitted data significantly better than a less parameter rich nested model that does not allow for positive selection.

Detecting amino acids under positive selection

To test for positive selection on the RCO protein sequence, we used likelihood ratio tests implemented in the PAML package (Yang et al. 2005; Yang 2007). To perform this analysis, the alignable part of the *RCO* promoters (directly upstream of ATG) and CDSs from 7 species (*A. halleri*, *A. lyrata*, *C. hirsuta*, *L. alabamica*, *S. irio*, *E. salsugineum*, and *Capsella rubella*) were aligned using MUSCLE and concatenated. The phylogeny was reconstructed using maximum likelihood with the Tamura-Nei substitution model (Tamura and Nei 1993), as implemented in MEGA software v6.06 (Tamura et al. 2013).

Robustness of the phylogeny was tested with 1000 bootstrap replicates. The analysis of selection was performed entirely on the *RCO* coding region (alignment included 118 codons). Maximum-likelihood analysis of the RCO data set was performed with codeml of the PAML software package, the alignment was fitted to F61 codon frequency models. We applied models of codon evolution that allow for variation in the ω ratio (dN/dS) among codons/amino acids in the protein but assume the same distribution in all branches of the phylogeny. We performed three likelihood ratio tests for positive selection: M1a-M2a and M7-M8. The M1a and M7 models are the null models without positive selection (no codons are allowed to have $\omega>1$) and the M2a and M8 models are the alternative models with positive selection. The significance of the LRTs was calculated assuming that twice the difference in the log of maximum likelihood between the two models is distributed as a chi-square distribution with two degrees of freedom (given by the difference in the numbers of parameters in the two nested models).

Cycloheximide treatment

Twelve day old seedlings grown onto MS plates in short days (8L: 16D) were incubated in liquid MS medium with either 0.1% DMSO or 300 μM cycloheximide. Seedlings were harvested by flash freezing in liquid nitrogen at the indicated time points.

Immunoblotting

For immunoblotting, protein extracts were prepared in extraction buffer [50 mM Tris-HCl (pH 7.5), 10% glycerol, 1 mM EDTA, 150 mM NaCl, 0.5% Igepal, 1mM PMSF, and protease inhibitor cocktail according to the manufacturer's instruction (Sigma-Aldrich; P9599) and following standardization of protein concentrations, subjected to size separation by SDS-PAGE and electrotransferred onto polyvinylidene fluoride membranes

(Millipore). RCO-3HA/RCOA48D-3HA and ACTIN levels were monitored with rat monoclonal anti-HA (clone 3F10) and mouse monoclonal anti-ACTIN (clone C4) antibodies, respectively. Immunocomplexes were detected by autoradiography of enhanced chemoluminescence generated by horseradish peroxidase-coupled goat anti-rat (Sigma-Aldrich; A 9037) and goat anti-mouse (Thermo Scientific Pierce; 31430) secondary antibodies. The immunoblot signals were quantified using the AlphaVIEW SA software (Cell Biosciences).

Photosynthesis and seed analysis

Plants were grown at the tested temperatures in single small pots (8 cm diameter) for 21 days, under long day-length conditions. At 14 and 21 days photosynthesis was assessed with LICOR LI 6400XT, with CO_2 and light values optimized for *Arabidopsis* (Long and Hällgren 1993). After each measurement, the rosettes of the plants analyzed were removed from the pot to take their silhouettes and calculate the rosette area. These values were used to normalize the CO_2 absorption rates. For experiments with *rco*, we used complemented *rco* mutant plants that looked indistinguishable to wild type plants as controls. The same genotypes were grown at the same conditions until flowering and seed production. Seed size and seed weight measurements were performed using the MARVIN set up from GTA Sensorik GmbH, with the exception of *A. thaliana* wild type and *RCOg* seed size, which was determined by photographing seeds on millimeter paper and determining size using ImageJ. Quantification of embryo and endosperm size was performed using ImageJ on DIC micrographs.

Post-embryonic *RCO* expression is restricted to the base of young leaf primordia, while in embryos it is only expressed transiently in a few cells at the base of cotyledons at the

torpedo stage (Vlad et al. 2014) (Supplemental Fig. 7A,B). Note that endosperm size, but not embryo size, is increased in *C. hirsuta* wild type and *A. thaliana RCOg* compared to the *rco* mutant and *A. thaliana* respectively at a stage before the onset of transient *RCO* expression (Supplemental Fig. 7C-E).

Supplemental references

Benfey PN, Ren L, Chua NH. 1990. Tissue-specific expression from CaMV 35S enhancer subdomains in early stages of plant development. *EMBO J* **9**: 1677-1684.

Clough SJ, Bent AF. 1998. Floral dip: a simplified method for Agrobacterium-mediated transformation of Arabidopsis thaliana. *Plant J* **16**: 735-743.

Dubchak I, Brudno M, Loots GG, Pachter L, Mayor C, Rubin EM, Frazer KA. 2000. Active conservation of noncoding sequences revealed by three-way species comparisons. *Genome Res* **10**: 1304-1306.

Frazer KA, Elnitski L, Church DM, Dubchak I, Hardison RC. 2003. Cross-species sequence comparisons: a review of methods and available resources. *Genome Res* **13**: 1-12.

Hay A, Tsiantis M. 2006. The genetic basis for differences in leaf form between Arabidopsis thaliana and its wild relative Cardamine hirsuta. *Nat Genet* **38**: 942-947.

Koncz C, Schell J. 2002. T-DNA tagging. in *Molecular Plant Biology* (eds. P Gilmartin, C Bowler), pp. 33-51. Oxford University Press, Oxford.

Long SP, Hällgren JE. 1993. Measurement of CO2 assimilation by plants in the field and the laboratory. in *Photosynthesis and Production in a Changing Environment: a field and laboratory manual*, pp. 129-167. Chapman & Hall, London, UK.

McLellan T, Endler JA. 1998. The relative success of some methods for measuring and describing the shape of complex objects. *Systematic Biology* **47**: 264-281.

Tamura K, Nei M. 1993. Estimation of the number of nucleotide substitutions in the control region of mitochondrial DNA in humans and chimpanzees. *Mol Biol Evol* **10**: 512-526.

Tamura K, Stecher G, Peterson D, Filipski A, Kumar S. 2013. MEGA6: Molecular Evolutionary Genetics Analysis version 6.0. *Mol Biol Evol* **30**: 2725-2729.

Vlad D, Kierzkowski D, Rast MI, Vuolo F, Dello Ioio R, Galinha C, Gan X, Hajheidari M, Hay A, Smith RS et al. 2014. Leaf shape evolution through duplication, regulatory diversification, and loss of a homeobox gene. *Science* **343**: 780-783.

Wong WS, Nielsen R. 2004. Detecting selection in noncoding regions of nucleotide sequences. *Genetics* **167**: 949-958.

Yang Z. 2007. PAML 4: phylogenetic analysis by maximum likelihood. *Mol Biol Evol* **24**: 1586-1591.

Yang Z, Wong WS, Nielsen R. 2005. Bayes empirical bayes inference of amino acid sites under positive selection. *Mol Biol Evol* **22**: 1107-1118.

Zhang J, Nielsen R, Yang Z. 2005. Evaluation of an improved branch-site likelihood method for detecting positive selection at the molecular level. *Mol Biol Evol* **22**: 2472-2479.

Supplemental Figure Legends

Supplemental Figure S1. Strong evolutionary conservation of *ChRCOenh*^{500}. Multiple alignment of *LMI1* and *RCO* 5' upstream sequence regions from all Brassicaceae species indicated on the y-axis to 3.2 kb of *C. hirsuta RCO* 5' upstream sequence on the x-axis revealed one region that is more strongly conserved among *RCO* genes than *LMI1* genes, denoted as "Region B", which corresponds to *ChRCOenh*^{500}. The alignment was constructed using the Shuffle-LAGAN algorithm with 70% identity threshold and 100 bp sliding window and visualized with the mVISTA browser. Pink shade denotes CNS (>70% identity over a 100 bp sequence length). The *A. lyrata LMI1* and *RCO* pairwise alignments to *C. hirsuta RCO* were used in Fig. 1B to explain our regionalization of the 5' upstream sequence (indicated by red stars).

Supplemental Figure S2. A 5' truncation of both the *ChRCO* and *ChLMI1* upstream sequence does not alter their characteristic expression patterns. **(A)** Schematic representation of GUS reporter constructs using the previously reported[10] *ChRCO* (1) and *ChLMI1* (3) upstream region and a 5' truncation of 1314 bp for *ChLMI1* (2) and 894 bp for *ChRCO* (4). The regions upstream of A^{LMI} and A^{RCO} were found to be dispensable for determining the specific expression patterns of their respective genes. **(B-G)** Representative GUS-stained leaves obtained from *A. thaliana* plants transformed with reporter constructs depicted in A and H. In each image the construct used is indicated (1-6). Stipules are indicated by a black star. Scale bar in B-E is 100 µm, the scale bar in F and G is 50 µm. **(h)** Schematic representation of GUS reporter constructs. *Aethionema arabicum LMI1* 5' upstream region (5) is depicted (blue) including enhancer sequence (orange). To show necessity of the enhancer for expression, we randomized its sequence within the *AetLMI1* 5' upstream context (6).

Supplemental Figure S3. The leaf phenotype of transgenic *A. thaliana* plants expressing *RCOY56S* is indistinguishable from plants expressing *RCO* while the A48D mutation has a major effect on leaf form.

Supplemental Figure S4. The RCOgA48D protein has a higher potency to rescue the *C. hirsuta rco* mutant than RCO. **(A)** Transgenic *rco* mutant lines harboring *RCOg* or *RCOgA48D* were categorized into four classes (Class I-IV) according to the severity of the leaf phenotype. Representative leaf 5 silhouettes shown. **(B)** Column graph shows that plants with highly dissected leaves (Class IV) are only found in the transgenic *rco* lines harboring *RCOgA48D* and the frequency of fully complemented *rco* mutants (Class I) in *rco* lines containing *RCOgA48D* is higher than in *rco* lines containing *RCOg*. **(C,D)**

Quantification of the leaf dissection index (C) and leaf area (D) of leaves 7-10 of *C. hirsuta* wild type and *rco*; *RCOgA48D* Class IV. A t-test was used to calculate significance. (*) $P < 0.05$; (**) $P < 0.01$; (***) $P < 0.001$. Error bars indicate standard deviation based on at least 15 T1 lines.

Supplemental Figure S5. Scanning electron micrographs of the adaxial laminal surface of fully expanded leaves in *A. thaliana* and *C. hirsuta*. The size of epidermal pavement cells in the plants expressing *RCOA48D* is smaller than their corresponding wild type. A t-test was used to calculate significance. (*) $P < 0.05$; (**) $P < 0.01$; (***) $P < 0.001$. Error bars indicate standard deviation based on at least 7 T1 lines.

Supplemental Figure S6. RCOA48D-3HA is more stable than RCO-3HA. **(A)** qRT-PCR comparison of *RCO* and *RCOA48D* transcript levels relative to *UBQ10* standard. The error bars represent standard deviation of triplicate qRT-PCR measurements using three biological replicates. **(B-C)** Quantitative analysis of RCO and RCOA48D proteins in three independent transgenic lines. Coomassie blue staining (a slice of SDS-PAGE gel containing a 29 KDa band) and immunoblotting with anti-ACTIN indicate equal loading of protein samples. The experiment was conducted twice and a t-test was used to calculate significance (N.S. = not significant, * $p<0.05$, ** $p<0.01$, *** $p<0.001$). Error Bars represent standard deviation.

Supplemental Figure S7. *RCO* influences seed endosperm size. **(A,B)** Fluorescent expression of a *pChRCO::ChRCO:Venus* translational reporter in *C. hirsuta* (A) and *A. thaliana* (B) torpedo stage embryos. Yellow arrowheads indicate *ChRCO:Venus* expression at the base of cotelydons. **(C)** Representative DIC micrographs of *C. hirsuta*

seeds used to calculate endosperm and embryo size. Coloured shading to indicate where the endosperm (magenta) and embryo (green) areas reside in the plant seed. **(D,E)** Quantification of embryo and endosperm size measured using ImageJ on DIC micrographs. A t-test was used to calculate significance (N.S. = not significant, * p<0.05, ** p<0.01, *** p<0.001). **(F)** Stomatal density in *rco* and *RCOg* mutants compared to their wild type counterparts. Leaf surfaces of the four genotypes were analyzed using agarose prints, stomatal number was determined per unit of leaf area and divided by the total leaf area. For each genotype 10 leaves were analyzed. A t-test was used to calculate significance. (N. S. = not significant, p > 0.05). The scale bar is 100 µm in (A-B) and 150 µm in (C). Error bars represent standard deviation.

Supplemental Table S1. RCO log likelihood scores and parameter estimates for four models of variable ω among sites assuming the f61 model of codon frequencies. ω=dN/dS. Bayes Empirical Bayes (BEB) analysis. Naive Empirical Bayes (NEB) analysis. The numbers of amino acids under positive selection (A49 and Y57) are according to the RCO protein sequence of *C. hirsuta* in the alignment. The true numbers of the amino acids according to the RCO protein sequence of *C.hirsuta* are A48 and Y56

Supplemental Table S2. Oligonucleotides used for quantitative real-time PCR.

Supplemental Table S3. Promoter swap constructs used in this study and analyzed in Fig. 1, Fig. 2 and Supplemental Fig. 2. The Constructs have been numbered according to the Fig. Numbered nucleotides in the table refer to the LMI1 or RCO promoter in accordance with the swapped fragment. Promoter sizes represent the upstream intergenic space, starting from the “ATG” of both LMI1 and RCO genes.

Supplemental Figures

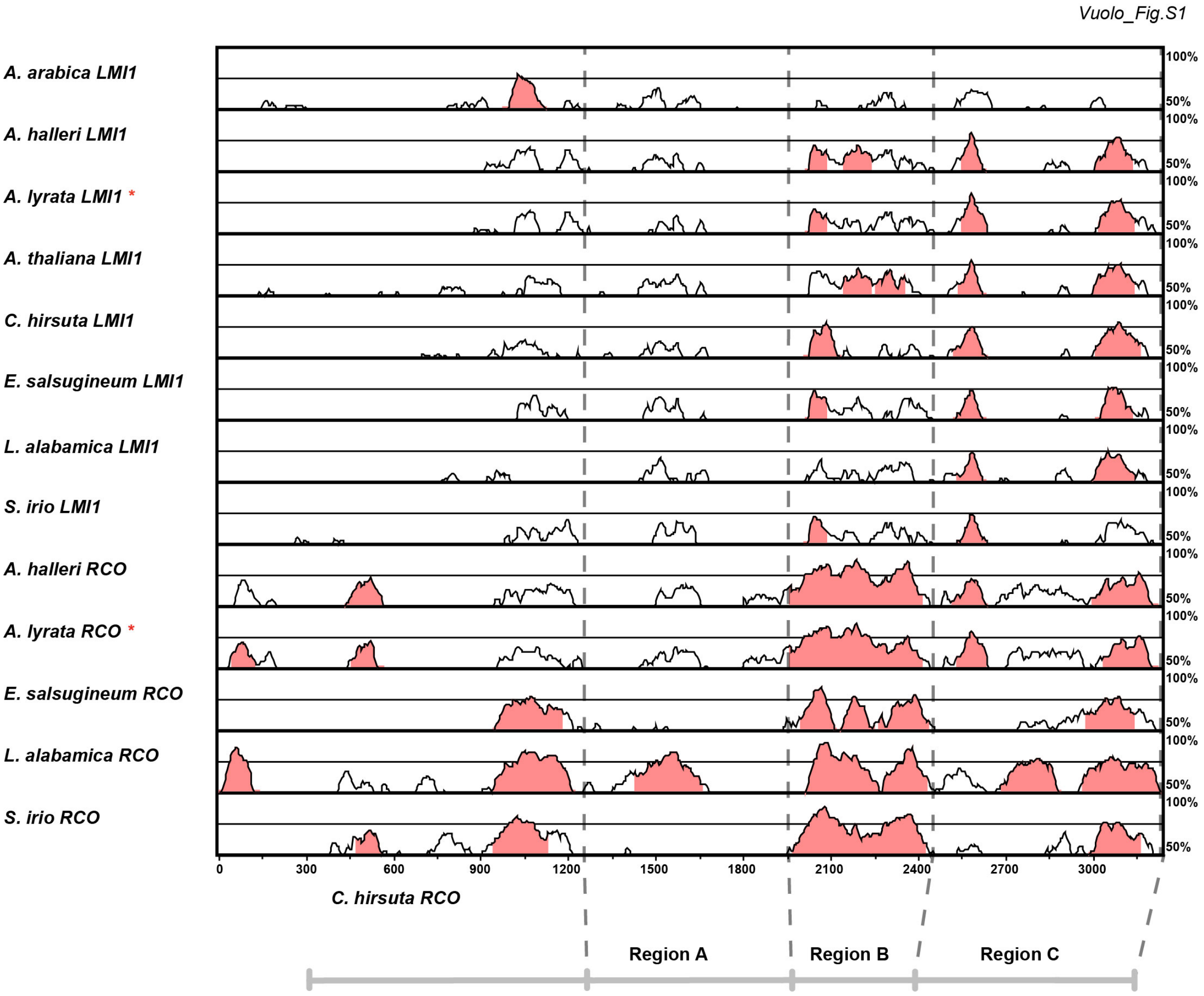

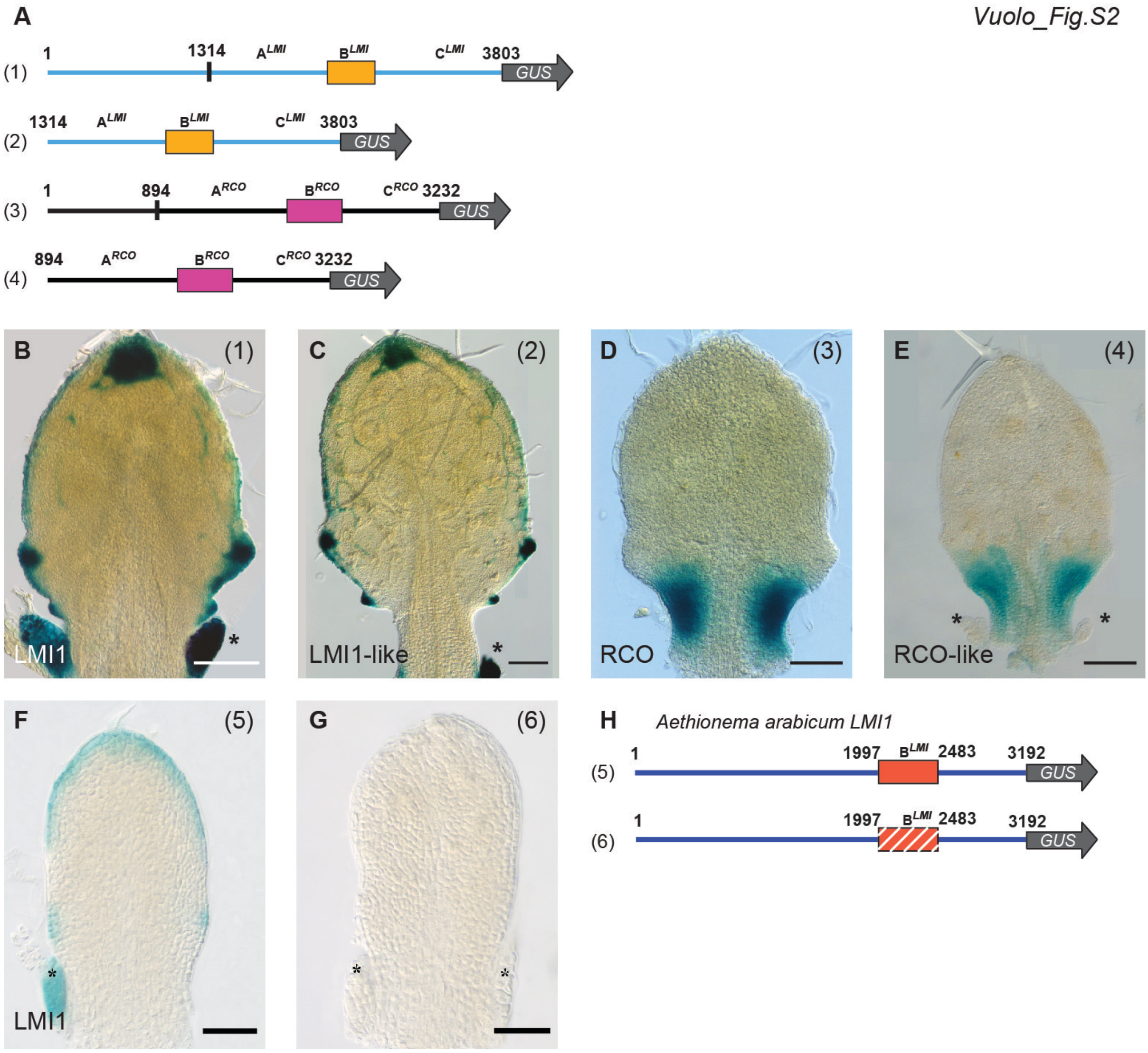
A
(1)
1
1314
A^LMI
B^LMI
C^LMI
3803
GUS
(2)
1314
A^LMI
B^LMI
C^LMI
3803
GUS
(3)
1
894
A^RCO
B^RCO
C^RCO 3232
GUS
(4)
894
A^RCO
B^RCO
C^RCO 3232
GUS
B (1)
LMI1
C (2)
LMI1-like
D (3)
RCO
E (4)
RCO-like
F (5)
LMI1
G (6)
H Aethionema arabicum LMI1
(5)
1
1997
B^LMI
2483
3192
GUS
(6)
1
1997
B^LMI
2483
3192
GUS

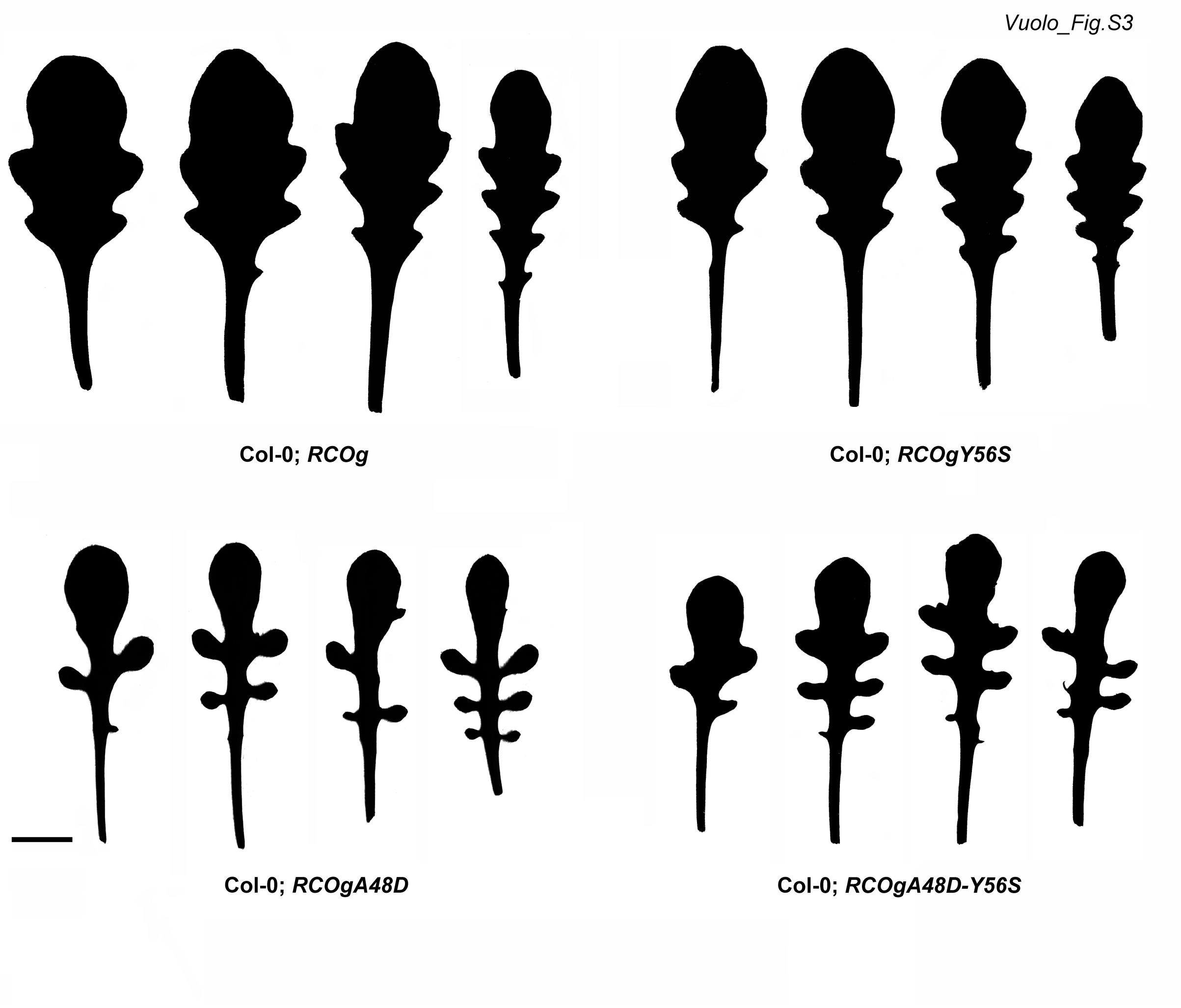
Vuolo_Fig.S3
Col-0; *RCOg*
Col-0; *RCOgY56S*
Col-0; *RCOgA48D*
Col-0; *RCOgA48D-Y56S*

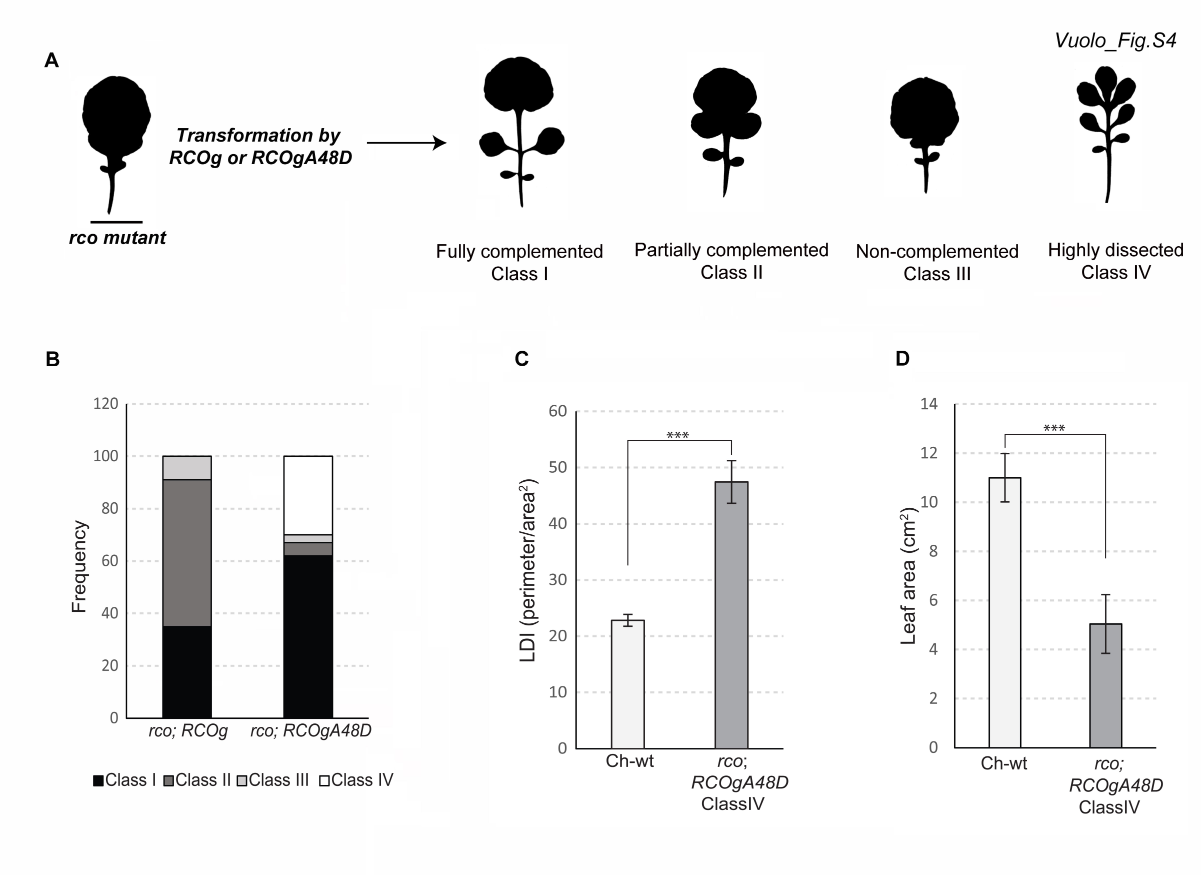
Vuolo_Fig.S4
A
Transformation by
RCOg or RCOgA48D
rco mutant
Fully complemented
Class I
Partially complemented
Class II
Non-complemented
Class III
Highly dissected
Class IV
B
Frequency
rco; RCOg
rco; RCOgA48D
Class I
Class II
Class III
Class IV
C
LDI (perimeter/area²)
***
Ch-wt
rco;
RCOgA48D
ClassIV
D
Leaf area (cm²)
***
Ch-wt
rco;
RCOgA48D
ClassIV

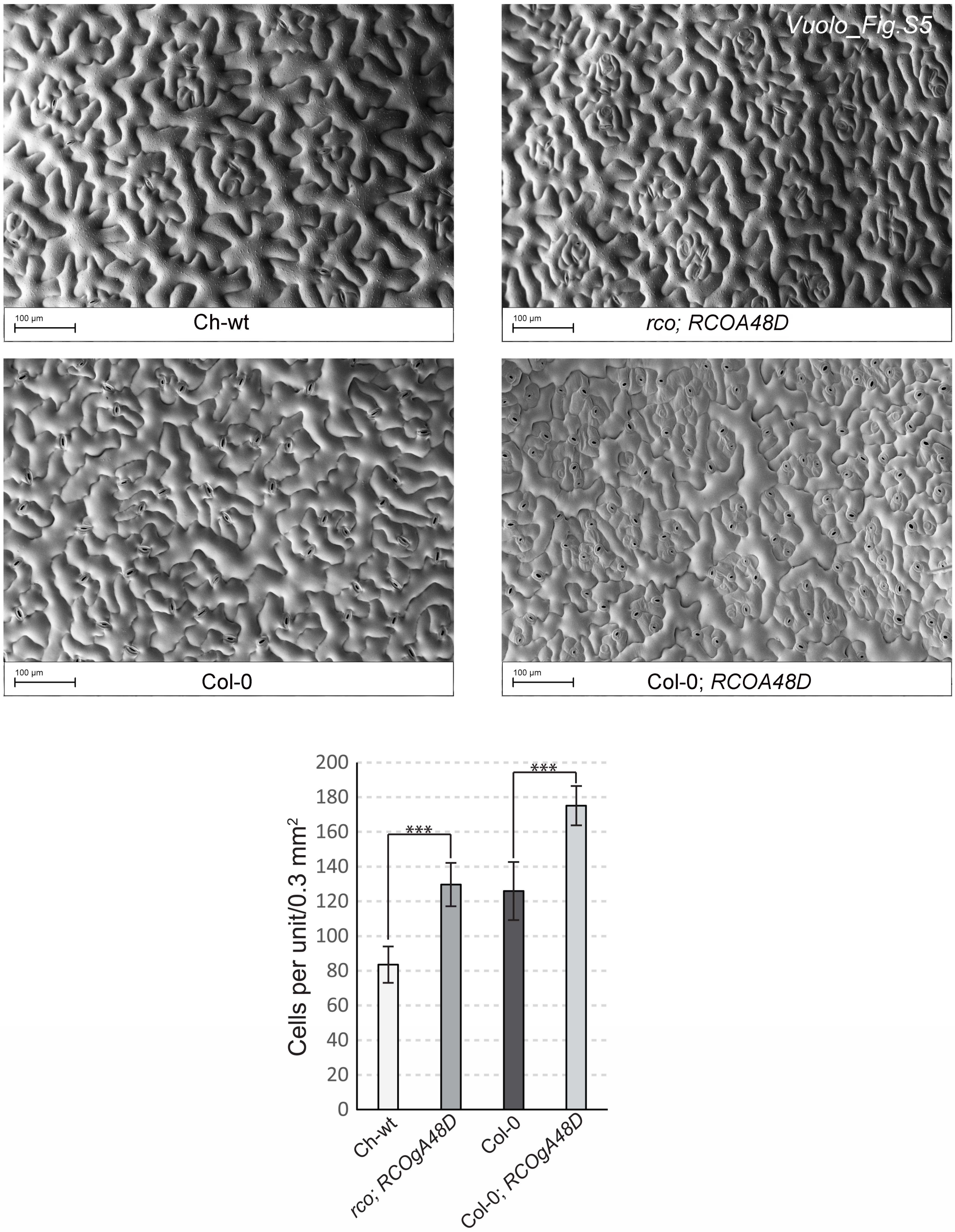

Vuolo_Fig.S5
100 µm
Ch-wt
100 µm
rco; RCOA48D
100 µm
Col-0
100 µm
Col-0; RCOA48D
Cells per unit/0.3 mm²
200
180
160
140
120
100
80
60
40
20
0
***
***
Ch-wt
rco; RCOgA48D
Col-0
Col-0; RCOgA48D

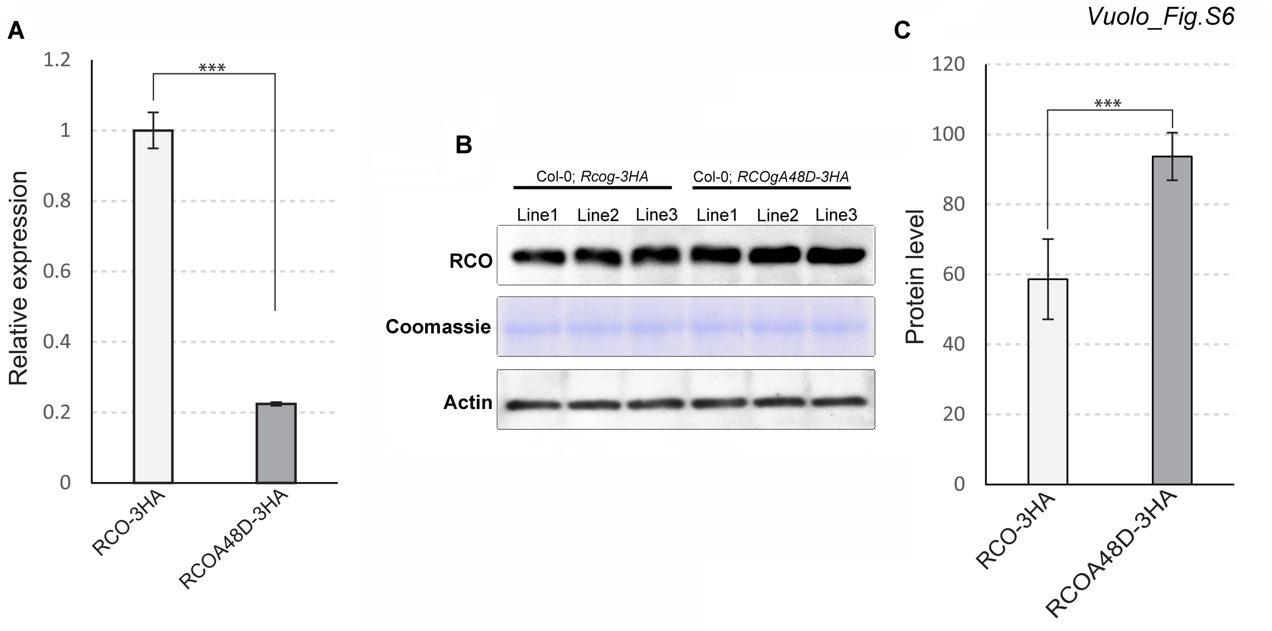
Vuolo_Fig.S6
A
Relative expression
***
RCO-3HA
RCOA48D-3HA
B
Col-0; Rcog-3HA
Col-0; RCOgA48D-3HA
Line1
Line2
Line3
RCO
Coomassie
Actin
C
Protein level
***

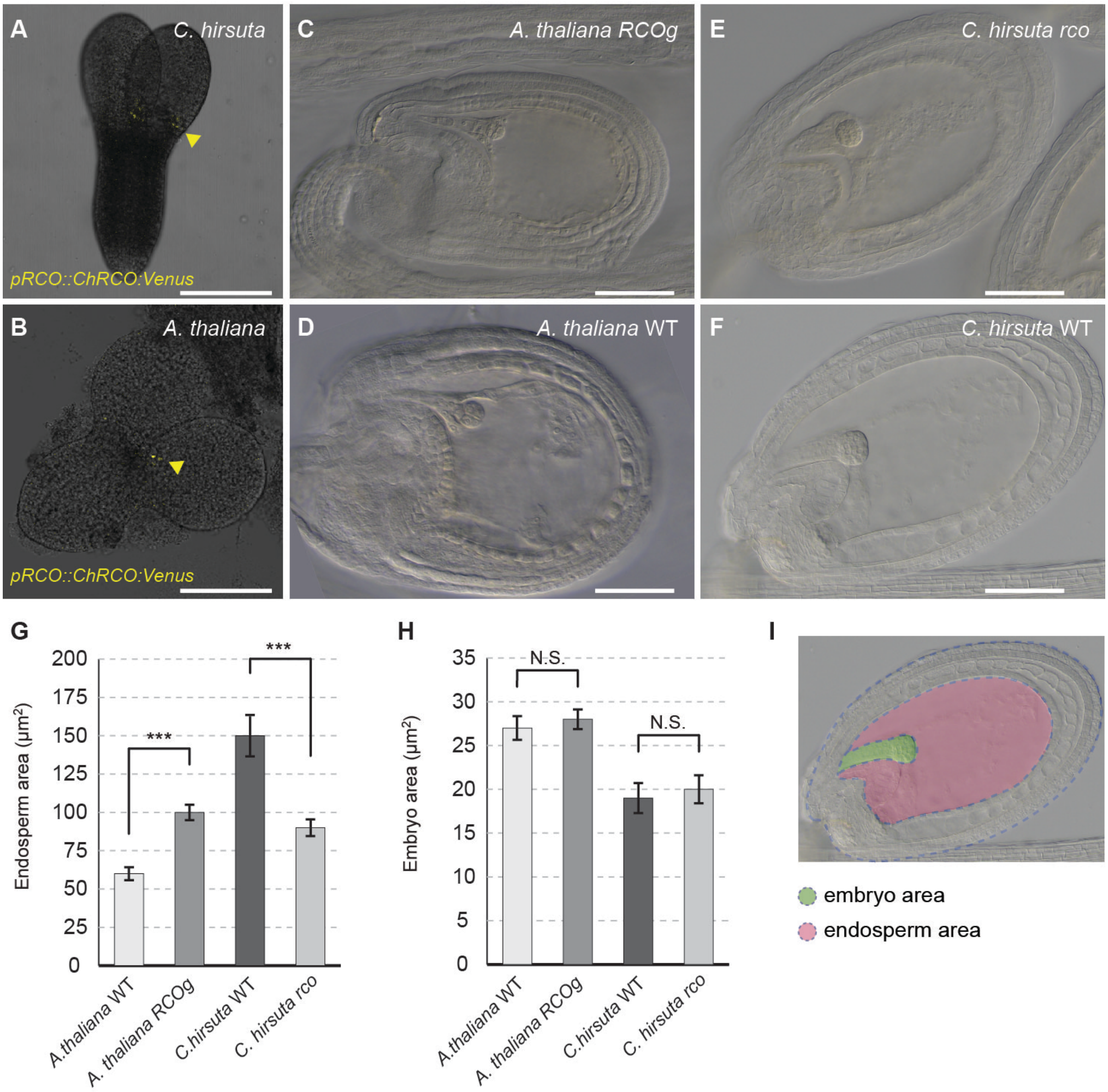
A
C. hirsuta
pRCO::ChRCO:Venus
B
A. thaliana
pRCO::ChRCO:Venus
C
A. thaliana RCOg
D
A. thaliana WT
E
C. hirsuta rco
F
C. hirsuta WT
G
Endosperm area (µm²)
200
175
150
125
100
75
50
25
0
***
***
A.thaliana WT
A. thaliana RCOg
C.hirsuta WT
C. hirsuta rco
H
Embryo area (µm²)
35
30
25
20
15
10
5
0
N.S.
N.S.
A.thaliana WT
A. thaliana RCOg
C.hirsuta WT
C. hirsuta rco
I
embryo area
endosperm area

Supplemental Tables

Vuolo_Table.S1

Site model	LnL	LRT* value	LRT* *P*	LRT* df	Type of selection	Parameter estimates** proportion of codons	Parameter estimates** omega	Sites (Pr[omega>1])*** NEB analysis	Sites (Pr[omega>1])*** BEB analysis
M1a	-1411,8762				purifying	**0,766**	**0,239**		
					neutral	*0,234*	*1*		
		6,433	<0.05	2	purifying	**0,835**	**0,307**		
M2a	-1408,6596				neutral	**0,136**	*1*	A49 (0.951)	A49 (0.939)
					adaptive	*0,029*	**4,775**	Y57 (0.953)	Y57 (0.944)
M7	-1413,5727				purifying	*1*	beta (p=**0.77**, q=**0.99**)		
		10,14	<0.01	2	purifying	**0,968**	beta (p=**2.09**, q=**3.25**)	A49 (0.986)	A49 (0.967)
M8	-1408,5023				adaptive	*0,032*	**4,681**	Y57 (0.987)	Y57 (0.970)

* Likelihood ratio tests for M1a/M2a and M7/M8 model comparisons

**Estimates for free parameters are in bold; values that are not free parameters (e.g. because proportions sum to 1) are shown in italic

*** Sites where adaptive selection was detected with Naïve Empirical Bayes (NEB) and Bayes Empirical Bayes (BEB) approaches

Vuolo_Table.S2

**Gene**	**Primer**	**Sequence**
RCO	RCOF	GGTCAGGGTTTAATGAAGACGCAAACAAATC
	RCOR	GGAAAAGCCTGAGATATCGCCG
UBQ10	UBQF	TGGTACTTTCGTGTGTTTTGAGGC
	UBQR	AAAGAGAGATAAGGACGCAAACATAGT

Vuolo_Table.S3

Construct	Description
**Supplemental Fig. 2** Construct 1	Full length LMI1 promoter (1 to 3803) driving GUS gene.
**Supplemental Fig. 2** Construct 2	5’ truncated LMI1 promoter (1314 to 3803) driving GUS gene.
**Supplemental Fig. 2** Construct 3	Full length RCO promoter (1 to 3232) driving GUS gene.
**Supplemental Fig. 2** Construct 4	5’ truncated RCO promoter (894 to 3232) driving GUS gene.
**Fig. 1** Construct 1	RCOp from its 894th nt to its 1954th nt – LMI1p from 2282nd nt until its 3803rd nt, driving GUS gene.
**Fig. 1** Construct 2	LMI1p from its 1314th nt to its 2280th nt – RCOp from its 1955th nt to its 3232nd nt, driving GUS gene.
**Fig. 1** Construct 3	LMI1p from its 1314th nt to its 3277th nt – RCOp from its 2694th nt to its 3232nd nt, driving GUS gene.
**Fig. 1** Construct 4	RCOp from its 894th nt to its 2693rd nt – LMI1p from its 2838th nt to is 3803rd nt, driving GUS gene.
**Fig. 1** Construct 5	RCOp from its 894th nt to its 1954th nt – LMI1p from its 2281st nt to its 2837th nt – RCOp from its 2584th nt to its 3232nd nt, driving GUS gene.
**Fig. 1** Construct 6	LMI1p from its 1314th n to its 2280th nt – RCOp from its 1955th nt to its 2483rd nt – LMI1p from its 2838th nt to its 3803rd nt, driving GUS gene.

**Fig. 1** Construct 7	LMI1p from its 2281st nt to its 2837th nt - CAMV35S minimal promoter, driving GUS gene.
**Fig. 1** Construct 8	RCOp from its 1955th nt to its 2483rd nt – CAMV35S minimal promoter, driving GUS gene.
**Fig. 2** Construct 1	Full length RCO promoter driving RCO cds.
**Fig. 2** Construct 2	LMI1p from its 1314th n to its 2280th nt – RCOp from its 1955th nt to its 2483rd nt – LMI1p from its 2838th nt to its 3803rd nt, driving RCO cds.
**Fig. 2** Construct 3	RCOp from its 1955th nt to its 2483rd nt – CAMV35S minimal promoter, driving RCO cds.
